# 70-kDa heat shock cognate protein expression as a putative biomarker of heavy-metal burden in *Mytilus galloprovincialis*

**DOI:** 10.1186/s40064-015-1007-6

**Published:** 2015-07-14

**Authors:** Ivana Ratkaj, Paula Žurga, Aleksandar Bulog, Jasna Peter-Katalinić, Sandra Kraljević Pavelić

**Affiliations:** Department of Biotechnology, University of Rijeka, Radmile Matejčić 2, 51000 Rijeka, Croatia; Teaching Institute for Public Health of Primorsko-goranska County, Krešimirova 52a, 51000 Rijeka, Croatia

**Keywords:** Heavy metals, *Mytilus galloprovincialis*, HSC-70

## Abstract

**Background:**

Heavy metals naturally occur in the marine environment and ecosystems. Due to anthropogenic influence they became common waters and coastal regions pollutants in particular where their concentrations remain hazardously high. We therefore tested a protocol for combined analysis of 6 heavy metal (Pb, Cd, Cr, Zn, Fe and Hg) concentrations in mussels *Mytilus galloprovincialis* collected from a coastal industrial zone (shipyard locality) and mariculture facilities in combination with expression analysis of multi xenobiotic resistance related genes and stress-related gene (*HSC-70*).

**Findings:**

In this paper we tested a protocol for heavy-metal levels assessment by use of a highly sensitive analytical method, ICP-OES, combined with expression analysis of multi xenobiotic resistance related genes, including the stress-related gene encoding 70-kDa heat shock cognate protein on mussels (*Mytillus Galloprovincialis)*. Mussels from the shipyard locality had higher heavy metal concentrations, except Fe. Higher metal concentrations did not influence expression of multi xenobiotic resistance related genes with exception of stress-related gene (*HSC-70*) encoding 70-kDa heat shock cognate protein.

**Conclusions:**

Our results indicate that mussels sampled in the industrial area have increased metal concentrations in comparison with the aquaculture locality, that are accompanied by increased transcript levels of *HSC-70*.

## Introduction

Heavy metals naturally occur in the marine environment and ecosystems. Due to excessive anthropogenic influence, they have been growingly recognized as important factors underlying pathogenesis of several diseases. Indeed, they are amongst most common pollutants of waters in general (Spada et al. 2013). They are non-degradable and occur in forms or concentrations that have harmful effects on living organisms (Maanan 2007). In particular, increased concentrations of heavy metals, highly hazardous for marine ecosystems are being reported in coastal regions that are increasingly exploited for fishing and mariculture in (Spada et al. 2013). Therefore, there is a growing concern about health risk related to increasing heavy metals presence in the marine environment and consequently in marine organisms for food consumption (Annicchiarico et al. 2010). It is expected that development of -omics methods applied to environmental research will provide improved and more sensitive protocols or models for assessment of environmental issues, *i.e.* identification of environmental biomarkers related to health (García-Sevillano et al. 2013). A recently recognized model system for heavy metal contamination assessment is the marine mussel-based system due to mussels’ ability to accumulate heavy metals and thus, reflect the environmental pollution (Fang et al. 2003). Previous studies have shown that expression of multi xenobiotic resistance (MXR)-related genes and those that code for heat-shock proteins (*HSP*) family genes, might be used as indicators of environmental stress caused by contaminants (Franzellitti and Fabbri 2005; Luedeking and Koehler 2004). Herein, we present a proof-of concept protocol for combined assessment of heavy metal pollution and environmental stress in the coastal region or coastal mariculture facilities by use of the mussel *Mytilus galloprovincialis* system as a general bioindicator of environmental pollution.

## Material and methods

### Samples and biometry

Mussels *Mytilus galloprovincialis* were collected in the spring period of 2013 at two locations in Croatia, including the facility for mussel production in Limski kanal - Istria (a total of 15 mussels) and the industrial zone, with increased heavy-metal pollution, of the Third May Shipyard –Rijeka (a total of 15 mussels). Mussels were immediately transported into laboratory in plastic containers filled with 1 L of sea water per 1 mussel. Biometric analyses were conducted according to Crosby and Gale (Crosby and Gale 1990). Briefly, mussel’s height, width and length were measured and, after dissection, soft tissue was removed, weighted and dried at 60 °C for 48 h. Condition index (CI) was calculated from weight of dried tissue and internal capacity of a shell according to the formula IC = mass of dried tissue (g) ×1000/capacity of a shell (g). Final biometry data is presented as a mean value ± standard deviations.

### Analysis of heavy metals

The dried soft tissues were pooled according to the location of mussel’s origin and two pooled samples were made: industrial pollutant region sample and cultivating location sample. Those samples were further dried at 60 °C to constant weight and homogenised to a powder. For microwave digestion of samples, needed for the analysis of lead, cadmium, chromium, iron and zinc, three aliquots of approx. 0,5 g were weighed from each sample. For mercury analysis, where no digestion was needed, three aliquots of approx. 0,2 g were weighed from each sample. All results were expressed on a dry matter basis and presented as mean values ± standard deviations.

The samples were digested in the Anton Paar Multiwave 3000 microwave system (Perkin Elmer, USA) equipped with pressurized vessels, using 5 mL of 65 % nitric acid per sample (HNO_3_ Suprapur, Merck, Germany), over a 20 minutes operation cycle at 200 °C. The digested samples were then transferred to 25 mL volumetric flasks and ultrapure water (Siemens) was added to the mark.

The concentrations of six metals were determined. Lead (Pb), cadmium (Cd), chromium (Cr), zinc (Zn) and iron (Fe) were determined using the ICP-OES Optima 8000 equipped with S10 autosampler (Perkin Elmer, USA). The determination of total mercury (Hg) was performed using the mercury analyzer AMA254 (Leco, USA). Analytical blanks were prepared and run in the same way as the samples. The concentrations of metals were determined using external standards, with standard solutions prepared in the same acid matrix. Standards for the instrument calibration were prepared on the basis of multielement certified reference solution for ICP (Perkin Elmer, USA) and single element certified reference solution for mercury (LGC Standards, USA). The method for ICP-OES was validated using the IAEA-407 reference material (fish tissue), (International Atomic Energy Agency, Austria). Mean recoveries for Pb, Cd, Cr, Zn and Fe were 93,1 %, 103 %, 90,1 %, 94,3 % and 91,4 %, respectively. The method for total mercury was validated using the Standard Reference Material (mussel tissue), NIST 2976. Mean recovery for total mercury was 104,4 %. The detection limits for the methods were (μg/L): Pb 3; Cd 0,5; Cr 0,9; Fe 1,5; Zn 9; Hg 0,08. Certified Reference Material and procedures were used to evaluate trueness and repeatability of the analyses.

### Gene expression analysis

Total RNA was isolated from the foot of 30 mussel by RNeasy spin columns (Qiagen) according to manufacturer’s recommendations and 20 ng of RNA was reversely transcribed into cDNA by High Capacity cDNA Reverse Trancsription Kit (Applied Biosystems, Life technologies, USA) on GenAmp PCR System 2400 (Applied Biosystems, Life technologies). Expression of *s18* (housekeeping gene), *Mdr*, *Mvp*, *Pgp* and *HCP-70* genes was determined with primers and fluorescent FAM labeled probes (Sigma-Aldrich, Sigma Life Science) listed in Table 1 on iCycleriQ real time PCR Detection System (Bio-Rad, USA). All individual samples were analysed in triplicate and analysis of gene expression in mussels from polluted shipyard locality was compared with gene expression in mussels from the production facility (control samples) by use of standard software Rest 2009 V2.0.13. Obtained results for each group were combined and results are presented as average fold change. Statistically significant changes for fold change >2 (at p < 0.05) was calculated.

**Table 1:** **Detailed list of primer pairs and labelled probes used in the qPCR reaction**

Gene	Sequence 5ʹ - 3ʹ	FAM labelled probe	Conditions
**s18** ^**a**^	**For** GGTACGTGATATGCCTAC	CCTGCTGCCTTCCTTGGATG	53 °C
	**Rev** CCAGGAGTGGGTAATTTG		
**Mvp**	**For** GAAGGAGACTATTGACAGA	TCTTCTTCACCAGCCATTCCTCA	53 °C
	**Rev** CCACAACTTCTTCATAAGC		
**Pgp**	**For** AACGCACATGACTTCATA	CTCTGGCAATGGCTACTCGTT	53 °C
	**Rev** CAGGATTCTTGGGTCTCTA		
**Mdr**	**For** GCCTTTGTGATTGGTTTA	ATCTCCTCCTTCAGAACAATGGTGAT	53 °C
	**Rev** GACAGATTAATTGTCGTTGA		
**HSC-70**	**For** GACTCTCTGTTTGAAGGA	TACAAGCATCACAAGAGCCAGGT	53 °C
	**Rev** CATGGTTCCTCTGAAAAG		

Housekeeping gene is marked with an asterisk (^a^).

## Results and discussions

**Figure 1:**
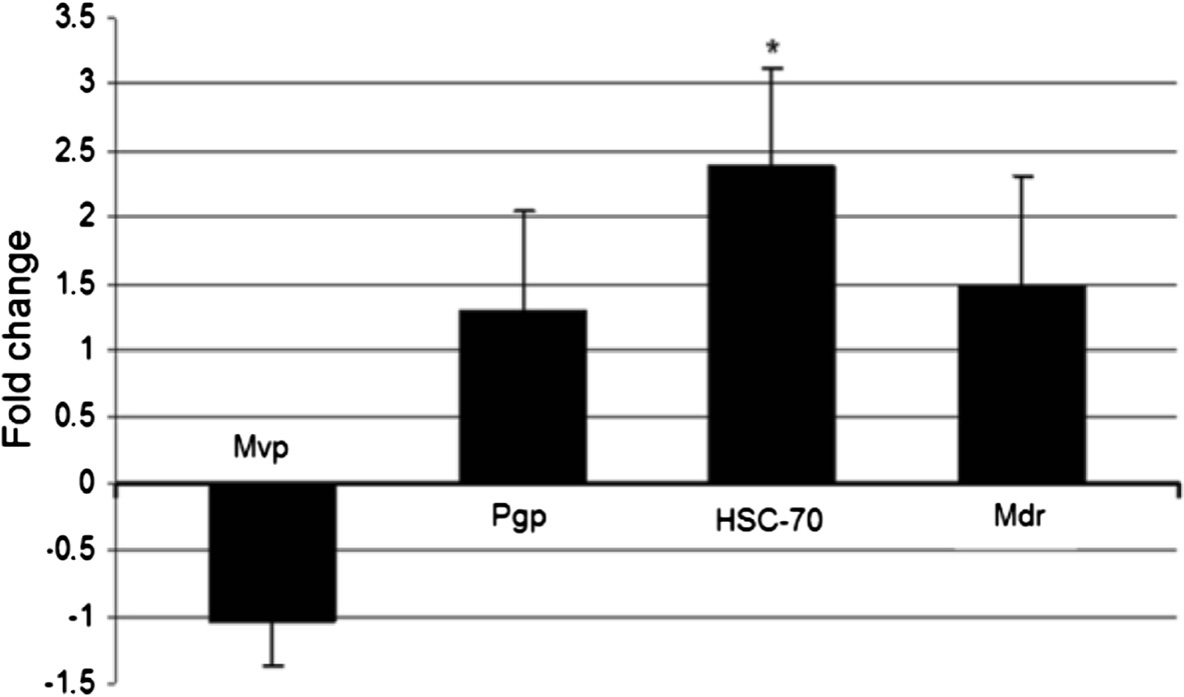
Average expression of stress-related gene encoding 70-kDa heat shock protein (*HSC-70*) and multi xenobiotic resistance-related transporters *Mvp* (major vault protein), *Pgp* (permeability glycoprotein) and *Mdr* (multidrug-resistance gene) in mussels collected from polluted region in comparison with mussels from the cultivating location. Statistically significant change (fold change > 2, p < 0.05) is marked with an asterisk (*).

Heavy metal contamination has been recognized as one of the most persistent problem in ecosystems that ultimately influences human health due to bioaccumulation of metals in the food chain (Nandi et al. 2012). The main goal of this paper was therefore, to test a combined protocol on mussels (*Mytillus Galloprovincialis)* as bioindicators of heavy metal accumulation and accompanying stress for assessment of heavy-metal pollution in a polluted shipyard locality. For that purpose we used a combined protocol for analysis of stress-related gene (*HSC-70*), multi xenobiotic resistance (MXR)-related genes’ status and six heavy metal concentrations in mussels from a chosen location with predicted heavy-metal pollution (shipyard location) in comparison with cultivated mussels grown in an ecologically stable unpolluted environment for mariculture. Biometric analysis revealed that mussels from both locations exhibited similar condition parameters including length, width and height (Table 2). Biometric similarity points to relatively uniform general environmental conditions at both localities. Importantly, higher metal concentrations were measured in mussels collected in the industrial zone. These were four times higher for lead and zinc, three times higher levels for chromium and two times higher for mercury in comparison with levels measured in cultivated mussels (Table 3). Iron concentration was higher in cultivated mussels probably due to greater local availability in the ecosystem which is non-toxic *per se* (George and Coombs 1977; Szefer et al. 2004). High lead concentrations are probably due to exposure to industrial discharges or city waste-water systems in the shipyard locality. Moreover, zinc overload in mussels might be also a result of larger available zinc quantities released from sediments disturbed by boat traffic. Having in mind that increased metal concentrations were assessed in the shipyard locality, we expected to measure higher expression levels of MXR-related genes involved in the detoxification system as well (Châtel et al. 2012; Kurelec 1997). However, no significant changes in expression of MXR-related genes in mussels with higher metal levels were observed (Fig. 1). On the other hand, the stress-related gene encoding 70-kDa heat shock cognate (*HSC-70*) protein transcript proved to be statistically increased in mussels from the shipyard locality. Previous research (Franzellitti and Fabbri 2006) has similarly provided evidence for increased *HSC-70* transcript expression after 8 h-exposure of mussels to CH_3_Hg^2+^ while exposure to Hg^2+^ alone induced increased *HSC-70* expression only after 6 day-treatment. Similar induction of the *HSC-70* gene was observed in mussels exposed to Hg^2+^ and Cr^6+^ (Franzellitti and Fabbri 2005). This might explain increased transcript levels of *HSC-70* in mussels with higher total metal concentrations in the presented study as well. *HSP70* is the most abundantly expressed proteins among the HSP family and has a constitutive expression in unstressed conditions (Hartl and Hayer-Hartl 2002). Changes in the mussel environment might often cause its over-expression (Franzellitti and Fabbri 2005; Piano et al. 2005). *HSC-70* is however, less stress-dependent in comparison with other stress-related genes and its role might be rather specialized, *i.e.* in long-term cytoprotection against inorganic metal contamination (Franzellitti and Fabbri 2006; Ali et al. 1996). We believe that observed alterations in *HSC-70* gene expression in mussels from the shipyard locality with higher metals level provide additional evidence on the sensitivity of *Mytillus galloprovincialis* to environmental changes*,* such as for example metal pollution as metal levels in analysed mussel specimens pointed only to moderately polluted waters. Moreover, we have shown that tested combined protocol for analysis of heavy-metal levels and activation of *HSC-70* gene in edible mussels (*Mytillus galloprovincialis)* might be used for efficient biomonitoring, especially in mariculture facilities. Still, presented study has some limitations that prevent us from more conclusive observations. In particular, a smaller number of specimens were analysed and only two localities were chosen for comparison of evaluated parameters. The same study design should therefore, be repeated on a larger number of specimens collected from various locations and at different seasons to achieve more reliable data and comprehensive conclusions.

**Table 2:** **Biometric parameters of analysed mussels with condition index**

Location	Condition index (CI)
**Industrial pollutant region**	**17,82 ± 8,52**
	length (cm): 6,04 ± 0,48
	width (cm): 3,48 ± 0,37
	height (cm): 2,11 ± 0,22
	mass (g): 27,72 ± 7,24
	shell mass (g): 11,64 ± 3,00
	dried tissue (g): 2,13 ± 1,45
	wet tissue (g): 7,89 ± 4,23
**Cultivating location**	**23,07 ± 8,24**
	length (cm): 6,40 ± 0,23
	width (cm): 3,37 ± 0,21
	height (cm): 2,16 ± 0,13
	mass (g): 27,48 ± 2,21
	shell mass (g): 9,60 ± 0,99
	dried tissue (g): 2,25 ± 1,00
	wet tissue (g): 4,92 ± 3,68

**Table 3:** **Concentration of six heavy metals (lead (Pb), cadmium (Cd), total mercury (Hg), chromium (Cr), zinc (Zn) an iron (Fe)) in mussel samples taken from the shipyard locality and cultivating location (results are expressed on a dry matter basis)**

Location	Pb (mg/kg)	Cd (mg/kg)	Total Hg (mg/kg)	Cr (mg/kg)	Zn (mg/kg)	Fe (mg/kg)
**Industrial pollutant region**	4,043 ± 0,222	1,278 ± 0,055	0,159 ± 0,005	2,827 ± 0,152	404,5 ± 15,6	116,8 ± 10,1
**Cultivating location**	0,995 ± 0,033	0,855 ± 0,031	0,079 ± 0,004	0,99 ± 0,026	117,5 ± 6,7	256,4 ± 18,4

## Abbreviations

MXR: Multi xenobiotic resistance
HSP: Heat shock protein
CI: Condition index
ICP-OES: Inductively coupled plasma optical emission spectrometry
HSC-70: 70-kDa heat shock cognate protein
Mvp: Major vault protein
PgP: Permeability glycoprotein
Mdr: Multidrug-resistance gene
